# Healthy Subjects With Extreme Patterns of Performance Differ in Functional Network Topology and Benefits From Nicotine

**DOI:** 10.3389/fnsys.2019.00083

**Published:** 2020-01-10

**Authors:** Carsten Gießing, Stefan Ahrens, Christiane M. Thiel

**Affiliations:** ^1^Biological Psychology, Department of Psychology, School of Medicine and Health Sciences, Carl-von-Ossietzky Universität Oldenburg, Oldenburg, Germany; ^2^Research Center Neurosensory Science, Carl-von-Ossietzky Universität Oldenburg, Oldenburg, Germany; ^3^Cluster of Excellence “Hearing4all”, Carl-von-Ossietzky Universität Oldenburg, Oldenburg, Germany

**Keywords:** nicotine, individual drug effects, fMRI, outlier, attention, cholinergic, graph theory

## Abstract

Do subjects with atypical patterns in attentional and executive behaviour show different brain network topology and react differently towards nicotine administration? The efficacy of pro-cognitive drugs like nicotine considerably varies between subjects and previous theoretical and empirical evidence suggest stronger behavioural nicotine effects in subjects with low performance. One problem is, however, how to best define low performance, especially if several cognitive functions are assessed for subject characterisation. We here present a method that used a multivariate, robust outlier detection algorithm to identify subjects with suspicious patterns of performance in attentional and executive functioning. In contrast to univariate approaches, this method is sensitive towards extreme positions within the multidimensional space that do not have to be extreme values in the individual behavioural distributions. The method was applied to a dataset of healthy, non-smoking subjects (*n* = 34) who were behaviorally characterised by an attention and executive function test on which *N* = 12 volunteers were classified as outliers. All subjects then underwent a resting-state functional magnetic resonance imaging (fMRI) scan to characterise brain network topology and an experimental behavioural paradigm under placebo and nicotine (7 mg patch) that gauged aspects of attention and executive function. Our results indicate that subjects with an atypical multivariate pattern in attention and executive functioning showed significant differences in nodal brain network integration in visual association and pre-motor brain regions during resting state. These differences in brain network topology significantly predicted larger individual nicotine effects on attentional processing. In summary, the current approach successfully identified a subgroup of healthy volunteers with low behavioural performance who differ in brain network topology and attentional benefit from nicotine.

## Introduction

Previous clinical studies on the therapeutic use of nicotine and nicotinic agonists often failed to show a positive effect on cognition, attention and psychomotor functions in patients with neuropsychiatric disorders (Newhouse, 2019). Despite these failures in clinical studies, previous experimental results revealed significant nicotine effects on motor performance and attention (Heishman et al., 2010; Tregellas and Wylie, 2019). In addition, previous clinical studies documented an increased percentage of smokers in patients with a variety of psychiatric disorders, including attention-deficit hyperactivity disorder (ADHD) and schizophrenia (Pomerleau et al., 1995; Tregellas and Wylie, 2019). This was seen as further evidence for the effectiveness of nicotine to self-medicate attentional and emotional dysfunctions (Gehricke et al., 2007). However, further research is needed to reliably identify subjects who will benefit from drug treatment and to characterise the underlying neural mechanisms of individualised drug effects in order to support a theory-driven approach of cognitive enhancement (Nebert et al., 2003; Picciotto, 2003; Nebert and Vesell, 2004; Colzato, 2017; Vergara et al., 2017; Ding et al., 2019).

Studies investigating the effectiveness of drugs to improve cognition in healthy humans and patients with neurological and psychiatric disorders show large variability in drug responses (Cools and D’Esposito, 2011; Newhouse et al., 2012). These effects are often explained by an inverted U-shape dose-response relationship in which extreme concentrations of a given neurotransmitter, either too little or too much, are related to low cognitive performance (Newhouse et al., 2004, 2012; Cools and D’Esposito, 2011). Plenty of experimental evidence, mostly for the low-performance range, supports that differences in baseline neurotransmitter levels and/or cognitive performance predict beneficial effects of cholinergic or dopaminergic stimulation (Cools et al., 2008; Turner et al., 2017). For example, a recent study in patients with mild cognitive impairment and healthy controls revealed that the neural response to cholinergic stimulation in an episodic memory task—in both groups—depends on the integrity of the cholinergic system, gauged by acetylcholine esterase activity (Richter et al., 2018). There is also prior evidence that the effects of cholinergic stimulation can be predicted by patterns of neural activity in an undrugged state (Giessing et al., 2007). Despite these insights, individualised drug therapies are however still an unsolved field of investigation.

Considering the disappointing results in clinical studies and the large variability of nicotine effects, the current study aimed to identify individual behavioural and neurobiological characteristics that allow predicting the effectiveness of nicotine administration in healthy volunteers. Previous behavioural studies examined baseline dependent effects of pro-cognitive drugs and related improvements after drug application to one single measure of baseline performance (Perkins, 1999; Perkins et al., 2000; Abreu-Villaça et al., 2006). However, reduced levels of neurotransmitter activity at the lower end of the inverted U-shape dose-response curve are likely to have a different degree of impact on a set of cognitive functions, which need to be gauged with multivariate approaches.

We used a new methodological approach to identify subjects that deviate from the overall multivariate data structure in behavioural pre-tests of attention, executive function and impulsivity. Based on a robust principal component analysis (PCA) we identified the position of each subject within a multidimensional latent performance space in order to select healthy subjects that strongly deviate from the centre of the multivariate data structure. The approach identifies outliers in the multivariate performance space and may thus also detect subjects who are not necessarily characterised by an extreme value in the univariate distribution (Gnanadesikan and Kettenring, 1972). We suggest that these outliers with atypical behavioural patterns, marked by low attentional or executive performance or high impulsivity, show larger improvements in attentional processing following nicotine administration (Niemegeers et al., 2014). Further, we aimed to describe how this cognitive phenotype is related to pre-existing differences in functional brain network topology with graph theoretical measures. Graph theoretical measures allow the assessment of local and global individual differences in network organisation. Previous studies suggest that individual attentional performance is related to the integration of information during mental processing (Giessing et al., 2013; Schultz and Cole, 2016). Thus, we hypothesised that subjects identified as outliers will differ from non-outliers in their nodes’ contribution to the cohesiveness or integration of brain networks. A resting-state functional magnetic resonance imaging (fMRI) scan, which was performed prior to the pharmacological intervention, was used to compare different measures of nodal centrality as measures of local brain network integration between outliers and non-outliers. In a final step, graph theoretical measures of nodal centrality derived from resting-state fMRI were used to predict differences in behavioural drug responses. Thus, we hypothesised that interindividual differences in nodal centrality during rest are an important biomarker for behavioural nicotine effects.

## Materials and Methods

### Subjects

Thirty-nine healthy non-smokers with normal or corrected to normal vision and no history of psychological illness were measured. Four subjects were excluded from the analysis either due to performance rates significantly below chance level (*N* = 1), incomplete functional magnetic resonance imaging (fMRI) scanning (*N* = 1), extensive head movements (*N* = 1, see below) or ceiling effects within the behavioural pre-tests [*N* = 1 with a hit rate of *p* = 1 and false alarm rate of *p* = 0.004 (one false alarm)]. The remaining 35 subjects [21 female/14 male, mean age 23.49 (SD 2.71)] entered the multivariate outlier detection approach. From these 35 subjects, one subject was excluded from all further analyses due to an inconsistent outlier classification (see below) leaving 34 subjects. The study was conducted in accordance with the Declaration of Helsinki with ethics approval obtained from the Ethics Committee of the German Psychological Association (DGPs). All procedures were carried out with written informed consent of all subjects. Subjects received monetary compensation for participation.

### Design

Data were collected in four experimental sessions. In the first session, baseline cognitive performance was assessed with two tests of the CANTAB test battery (CANTAB^®^; Cambridge Cognition, 2018^1^) and additional self-reports including the Barratt Impulsiveness Scale (BIS 15; Spinella, 2007; Meule et al., 2011) to assess individual performance levels in attentional and executive functioning. In the following session, for each participant, an fMRI resting-state scan with 527 scans was obtained during which subjects lay quietly with open eyes for 13 min in an MR scanner. In the third and fourth session, participants received either an inactive placebo or a 7 mg transdermal nicotine patch (Niquitin^®^ Clear 7 mg, GlaxoSmithKline Consumer Healthcare GmbH., München, Germany). Following a drug administration time of 60 min after which the patch was removed, subjects performed a sustained attention task with distractor and switch trials (see below) for 24 min in the MR scanner. To avoid any risk of skin burn, the nicotine patch was removed before the participants went in the scanner (Kuehn, 2009). Data from this task was used to measure the behavioural benefit from nicotine administration, the fMRI data acquired during task performance will be reported elsewhere. The drug administration was counterbalanced over subjects in a double-blind administration scheme. The average duration between placebo and nicotine administration was 11 days (minimum 6 and maximum 32 days). The resting-state scan was acquired 1 day prior to the first intervention session.

### Behavioural Pre-tests for Assessment of Baseline Cognitive Performance

To detect subgroups of subjects within the range of healthy, young volunteers that show conspicuous cognitive performance and deviate in their attentional or executive functions, performance in two CANTAB tests namely the Rapid Visual Information Processing (RVP) and the Intra/Extradimensional Set Shift (IED) was analysed.

The RVP task is a measure of sustained attention in which subjects have to detect target sequences of digits (for example, 2–4–6) within a stream of digits in pseudo-random order. From this task, the following three outcome measures were estimated for each individual: RVP response time latency, RVP sensitivity to the target (A’), and RVP strength of trace required to elicit a response (B”). Two of these parameters, RVP A’ and RVP B”, derived from a nonparametric version of signal detection theory (Grier, 1971; Frey and Colliver, 1973; Sahgal, 1987; Li et al., 2015). Whereas RVP A’ gauges sensitivity towards targets irrespective of the subject’s tendency to respond, RVP B” measures the individual perceptual bias or tendency to respond given a certain amount of “signalness” (Frey and Colliver, 1973). A liberal response criterion and the tendency to respond despite low perceptual evidence have been related to impulsiveness in healthy subjects and ADHD patients (Jones and McIntyre, 1976; Rodriguez and Baylis, 2007). In order to avoid subjective self-reports, we used the RVP B” as an objective indicator of impulsive behaviour. To *post hoc* validate our approach, we correlated the RVP B” with the German short version of the BIS 15 (Spinella, 2007; Meule et al., 2011), a subjective self-report of impulsiveness. In addition to the RVP task, participants performed the IED, a test of executive function. This task tests rule acquisition and reversal learning where either the rewarded stimulus (intra-dimensional) or the rewarded stimulus dimension (extradimensional) changed. From this task the following three outcome measures were estimated for each individual: errors after intra-dimensional changes (IED intra-dimensional set shift errors), errors after extradimensional changes (IED extradimensional set shift errors), and total amount of errors (IED total errors).

The RVP and IED were selected as pre-tests since both tests were successfully used to detect behavioural changes following cholinergic manipulations, such as the administration of nicotine or cigarette smoking (Sahakian et al., 1989; Jones et al., 1992; Nesic et al., 2011). In addition, previous results also support a link between impulsiveness and nicotine effects (Hosking et al., 2014). ADHD patients with high impulsivity tend to show larger nicotine effects on response inhibition (Potter and Newhouse, 2004; Potter et al., 2012) and previous fMRI studies revealed the largest effects of nicotine on the BOLD signal during inhibitory control in individuals with high levels of impulsivity (Kasparbauer et al., 2019).

### Outlier Detection Approach

**Figure 1:**
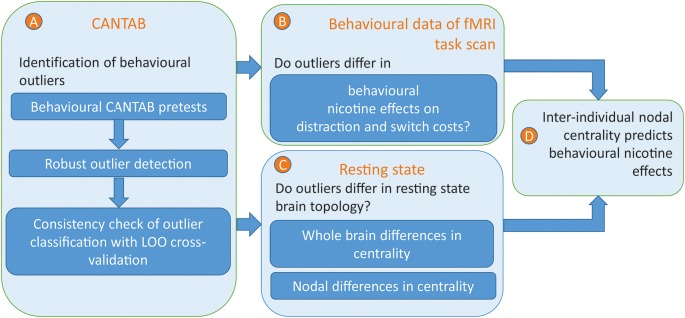
Overview of the analysis approach. **(A)** Behavioural outliers were identified by a robust outlier detection approach based on pre-measurements of attentional, executive, and inhibitory processing. To check the consistency of outlier classification, the outlier classification was repeatedly performed within a leave-out-one-sample approach to exclude subjects with unstable classifications. **(B)** On behavioural level, the effects of nicotine on distractor and switch costs were compared between outliers and non-outliers. **(C)** On a neural level, the brain network topology of outliers was compared with those of non-outliers. Therefore, three different measure of centrality were computed on the whole brain and nodal level. On the whole brain, it was investigated whether differences in centrality measures between outliers and non-outliers, either positive or negative, reflect systematic deviations over and above effects of noise. For those network densities and centrality measures that revealed a significant effect, the most significant effect was compared on nodal level. **(D)** Within those brain regions in which eigenvector centrality significantly differed between outliers and non-outliers, eigenvector centrality was correlated with the individual nicotine effects on distractor costs.

Performance in the behavioural pre-tests was analysed with an outlier detection procedure to identify subjects with atypical behavioural performance. To detect atypical performers, the outlier detection algorithm from Filzmoser et al. (2008) was used which is particularly effective in high dimensional data with few numbers of observations. The outlier detection was performed with the function “pcout” from the package “mvoutlier” in R statistics (The R Project for Statistical Computing^2^) with the pre-defined default values. Classical tools for outlier detection are often based on statistics like the mean and the covariance matrix that are however not suitable for data with outliers. In contrast, the current approach builds on robust statistics to avoid possible masking and swamping effects in which the classification of outliers depends on statistics like the mean which are heavily influenced by extreme values (Becker and Gather, 1999; Rousseeuw and Hubert, 2018). The principal components of performance measures in the pre-tests were computed, rescaled by subtracting the median and dividing by the median absolute deviation from the median (MAD). Following the default values, only those components were retained that explained at least 99 per cent of the variance (in our case five dimensions). For each subject, a robust Mahalanobis distance of the PCA scores from the median was computed to estimate the distance of each point from the centre of the multivariate distribution. The outlier detection algorithm of Filzmoser et al. (2008) uses two approaches to estimate two weights that reflect different aspects of “outlyingness.” Whereas the first approach aimed to detect location outliers, the second approach tried to detect scatter outliers. While location outliers result from a multivariate distribution with a different location parameter, scatter outliers possess a different scatter matrix. Both distance weights for location and scatter outliers were then integrated to an overall score to classify participants as outlier or non-outlier. The algorithm is described in detail in Filzmoser et al. (2008, pp. 9–14; see also Figure 1). To investigate the stability of the outlier classification, the outlier classification was performed *N* = 35 times with a leave-out-one-sample approach. Each time a different subject was excluded to investigate whether the outlier classification remained stable regardless of small deviations in the sampled data set. Subjects that were not consistently classified as outliers or non-outliers with a consistency of at least 90 per cent were excluded from the following analyses (*n* = 1, see above).

The following analyses were restricted to a specific subset of outliers within the multivariate PCA space to facilitate a functional interpretation of the observed neural differences. All outliers were selected and compared with the non-outliers that showed a PCA score in at least one of the first three PCA components that indicated, depending on the direction of the loadings, a worse performance or impulsive behaviour relative to the median. The first three components explained 97 per cent of the variance. Due to the relatively moderate sample size, the current analyses focussed on outliers within a relatively broad part of the multidimensional space. Future analyses with larger number of outliers might apply more conservative thresholds and restricted areas within the multivariate space to increase the homogeneity within the outlier group.

### Outlier Characterisation: Differences in Behavioural Pre-tests Between Non-outliers and Outliers

In multivariate data sets, outliers cannot be defined on the basis of each individual univariate distribution, since data points might only be extreme values within their multidimensional configuration. However, to facilitate a functional interpretation of how outliers differ from non-outliers we also plot the univariate distributions of the cognitive pre-tests and indicate the outliers’ positions.

### The Experimental Task to Assess Nicotine Effects

The sustained visual attention task with distractor and switch trials used in this study was adapted from Armbruster et al. (2012). Every 2 s subjects were presented with a stimulus for 900 ms. In 82% of the trials this was one digit above fixation and participants were asked to indicate with their right hand whether it was odd or even. Every 3–6 trials this ongoing task was interrupted by one of three conditions (each 6%). In these cases, another digit was presented below fixation and subjects were asked to respond to the brighter one. If the upper digit was brighter (distractor trial) the task rule was unchanged, if the lower digit was brighter (switch trial) subjects had to indicate using the left hand, whether this number was smaller or larger than 5. Assignment of task rules to the hands was counterbalanced across subjects. In the third condition (ambiguous trial; not analysed here) it was impossible to perceive brightness differences visually. There were in total 720 trials split up into six blocks of 120 with equal numbers of all conditions in every block. Blocks were separated by a pause of 20 s.

To assess individual effects of nicotine, the distractor costs of each subject were computed as the median response times during distractor trials minus median response times during ongoing trials. The corresponding calculation was performed for switch costs to assess performance in executive functioning (median response times during switch trials minus median response times during ongoing trials). In the following analyses, the effect of nicotine on distractor costs (distractor costs following nicotine administration minus distractor costs following placebo administration) were compared between outliers and non-outliers to investigate an “outlier by drug by distractor cost”-interaction. The corresponding contrast was also computed for switch trials to investigate an “outlier by drug by switch cost”-interaction.

### The Resting-State fMRI

#### Scanning Parameters

During the resting-state fMRI scans, T2*-weighted gradient echo-planar imaging (EPI) volumes (*N* = 527) with BOLD contrast were recorded by means of a 3-Tesla MRI scanner (Siemens Verio, Siemens AG, Erlangen, Germany): Repetition time (TR) = 1.5 s, echo time (TE) = 30 ms, flip angle (FA) = 80°, voxel size = 3 × 3 × 3.5 mm, between-slice gap = 1.05 mm, number of slices = 27 of 3.5 mm thickness. Structural T1-weighted images (MPRAGE sequence, TR 1.9 s, TE 2.52 ms, Voxel size 1 × 1 × 1, Flip angle 9°, 192 sagittal slices) were acquired in the same session as the resting-state fMRI scans.

#### Preprocessing

Preprocessing was performed with SPM12 (Wellcome Trust Centre for Neuroimaging, University College London; Frackowiak, 1997) and included removal of the first five images to account for possible T1 saturation effects, correction for head motion by spatially realigning functional images to the first volume, correction of timing differences between the slices and spatially normalisation to standard stereotaxic MNI space (Montreal Neurological Institute^3^). No spatial smoothing was applied to the images in order not to induce artificial correlations between time series which subsequently constitute the input data for the functional connectivity analysis.

#### Time Series Extraction and Movement Correction

Using a parcellated template from Shen et al. (2013), 217 brain nodes were defined covering cortical and subcortical brain regions (see also Finn et al., 2015). This template is based on a functional clustering approach that identifies functionally homogenous parcels that are consistent across subjects. For each parcel per subject, a mean fMRI time series was calculated.

#### Motion Artefact Removal

Rigorous movement corrections were applied. In a first step, following Power et al. (2012), framewise displacements (FD) were calculated for a standardised head with a radius of 50 mm and subjects who showed a maximum FD larger than 3.5 mm (*N* = 1) were excluded from the further analysis. In a second step, we used the DVARSCalc.m script from Afyouni and Nichols (2018) to identify and exclude brain scans in which the spatial root mean square of the data after temporal differencing (DVARs) significantly differed with a 5% familywise error rate^4^. On average, 3.16 per cent (±0.51 SEM) of the 522 scans were removed from each data set. Afterwards, the movement parameters from the realignment and six mean time series averaged across three-millimetre spheres within the white and grey matter volumes were regressed out from the fMRI time series. In the last step, regional time series were band-pass filtered with a Chebyshev Type 1 filter of order 8 within the range of 0.01–0.1 Hz (Biswal et al., 1995). Between each possible pair of regional time series, Fisher’s z-transformed Pearson’s correlations were computed. The absolute correlation matrix was thresholded to reduce possible effects of spurious and noisy connections. Since there is no golden standard for a single threshold, each absolute correlation matrix was repeatedly thresholded over a range of nine equally-spaced network densities with a minimum of 10 and maximum of 50 per cent density. Network density was defined as the number of edges divided by the maximum possible number of edges in the graph.

### Brain Network Topology Analysis

#### Computing Nodal Centrality Measures

Previous studies suggest that nicotine improves cognitive performance by changing the interaction between brain regions (Giessing et al., 2013). Thus, inter-individual differences in behavioural drug effects might be related to the baseline characteristics of brain networks before drug administration. Several studies suggest that efficient performance depends on the individual formation of processing pathways and the topology of large-scale brain networks (Wylie et al., 2012; Giessing et al., 2013). Therefore, a fundamental aspect to understand individual drug effects is to identify brain nodes that are central to propagate brain signals to a large portion of brain nodes (Newman, 2010; Malliaros et al., 2016). To investigate differences in nodal centrality between outliers and non-outliers, three measures of nodal centrality from graph theory were computed using the brain connectivity toolbox (Rubinov and Sporns, 2010; Fornito et al., 2016; Matas et al., 2017). As a first measure, “*betweenness centrality*” was calculated by the fraction of all shortest weighted paths in the network that contain a given node. Thus, nodes with high values of betweenness participate in a large number of shortest weighted paths between brain nodes. Second, we used the weighted undirected adjacency matrix to compute eigenvector centrality. *Eigenvector centrality* is a self-referential measure: the centrality score is proportional to the sum of the centrality scores of all nodes which are connected to it. Nodes get high eigenvector centrality if they are connected to other important nodes with high eigenvector centrality. If we aim the centralities to be non-negative, the eigenvector centrality of node i is equivalent to the ith element in the eigenvector corresponding to the largest eigenvalue of the adjacency matrix. As a last centrality measure, we computed the *k-coreness* of nodes to identify tightly interlinked groups within a network (Hagmann et al., 2008). To be part of the k-core of an undirected binary graph, a node has to be connected to at least k other nodes in the subgraph regardless of the connections to nodes outside this subgraph. The k-cores form a nested hierarchy, whereas nodes with highest k-coreness belong to the most interlinked, cohesive subgraph. For example, the three-core is the subset of nodes within the two-core that have at least three connections to all other members of the core. Thus, nodes with high k-coreness belong to tightly interlinked groups of nodes (Alvarez-Hamelin et al., 2005).

Comparing the different centrality measures, each measure gauges a slightly different aspect of node importance (Matas et al., 2017). *Eigenvector centrality* measures the connectedness of a node within a network; the eigenvector centrality of a node can be high because it is connected to many nodes or to nodes of high importance (or both). In contrast, *“betweenness centrality”* does not focus on the connectedness of a node, but how well a node controls flow between others by acting as a bridge along the shortest path between two other nodes. *k-coreness* is related to the propagation and spreading of information. The *k-core* decomposition progressively decomposes the networks in different layers revealing a nested structure of cores outmost to the most internal one. It has been shown that the nodes of the central core as identified by the k-shell decomposition analysis are most efficient in the spreading of information within the network. High efficient “spreaders” within the network do not necessarily correspond to the most highly connected nodes (Kitsak et al., 2010). In summary, the current analysis identifies different aspects of node centrality to investigate whether outliers and non-outliers differ in their most important nodes.

#### Outlier Effects on Brain Network Topology

Within the network topology analyses, networks with equal edge densities were compared between outliers and non-outliers (see above). This was done to distinguish effects on network topology from effects on network density (van Wijk et al., 2010). However, due to the fixed number of edges, an increase of nodal connectedness in one brain region likely correlates with a decrease of centrality in a different region. This would lead to small averaged effects, despite possible systematic, but heterogeneous and non-uniform patterns of effects across nodes. To investigate heterogeneous effects, previous studies suggested to report *sorted* individual effects in an increasing order and indexed by percentiles (Chernozhukov et al., 2018). Within a first approach on the global level, we aimed to test whether these differences between outliers and non-outliers, either positive or negative changes, reflect systematic deviations over and above effects of noise. Using a randomisation approach, we tested whether the sum of the squared differences between outliers and non-outliers was significantly larger than expected under the null hypothesis. Within this randomisation approach, outlier and non-outliers were randomly intermixed for 5,000 times and for each randomisation the squared differences were computed to estimate the null distribution. In the following nodal analyses, the network density with the highest significance level within the global analysis was selected and for each individual node it was tested whether the investigated measures of centrality significantly differed between outliers and non-outliers.

### Significance Testing

Statistical comparisons were performed with non-parametric, two-sided permutation tests with 5,000 permutations. On nodal level, alpha inflation due to multiple comparisons was controlled by the false discovery rate (FDR), the expected proportion of false discoveries amongst the rejected hypotheses (Benjamini and Hochberg, 1995). Permutation tests on nodal data used 100,000 permutations to estimate smaller *p*-values with higher accuracy.

## Results

### Identifying Atypical Performance in the Behavioural Pre-tests

**Figure 2:**
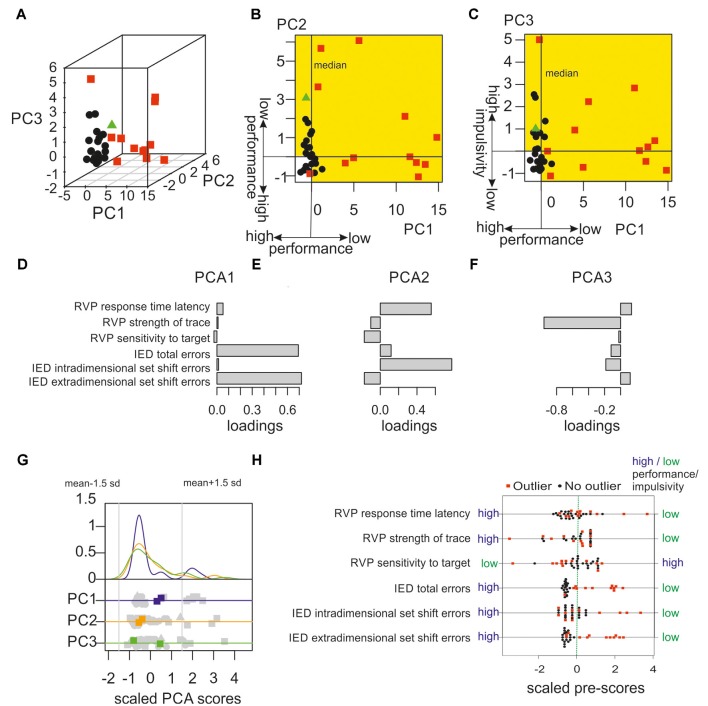
Identification of behavioural outliers with robust outlier detection. **(A)** An algorithm particularly effective in high dimensional data was used to detect extreme subjects in the behavioural pre-tests. In the first step, principal components of data were computed to group participants as non-outliers (black data points) or outliers (red data points). One data point (green triangle) was excluded from the following analysis due to inconsistent outlier classification (further details are given in the main text). **(B)** Illustration of the performance in the pre-tests showing the first vs. second PCA component. The following resting-state functional magnetic resonance imaging (fMRI) analysis only included outliers that showed PCA scores that were associated with lower performance or higher impulsivity in at least one of the first three PCA components (according to the loading patterns). All outliers fulfilled this criterion. Within the multidimensional space, the corresponding areas with reduced performance in comparison to median are colour-coded in yellow.** (C)** First vs. third PCA components of behavioural pre-tests are shown. **(D–F)** Loadings of the scaled behavioural variables (i.e., the variable weights or eigenvectors) for the first three components. Whereas the first component is mainly a linear combination of measures of executive functioning (i.e., Intra/Extradimensional Set Shift (IED) total errors and extradimensional set shift errors), the second component assessed both, executive functioning and sustained attention. The third component mainly gauges the subjects’ tendency to react following low perceptual evidence, a measure that correlates with impulsivity. **(G)** This plot illustrates that the multivariate outlier approach is more sensitive and detects more outliers in the current sample as a “traditional” univariate approach. Within the upper part the individual distributions of the PCA scores of the first (blue), the second (orange), and the third (green) component are illustrated with their corresponding density functions. In the lower part, scores of individual subjects on each PCA component are printed in grey. Multivariate outliers that were detected as outliers within the multivariate approach, but not within a univariate outlier definition are marked in blue, orange, and green, respectively. Univariate outliers were defined as data points 1.5 standard deviations below or above the mean in any of the first three PCA dimensions as indicated by the grey vertical lines. Data were normalised to mean zero and a standard deviation of one (square: outliers included in the data analysis; triangles: data points excluded due to inconsistent multivariate outlier classification; see above). **(H)** Outlier characterisation. Outliers were defined within a multidimensional space representing for each subject the main interindividual differences in six parameters of sustained attention and executive functioning. To further characterise outliers, the univariate distribution of these six behavioural parameters were plotted. Outliers tend to show worse performance in attention and executive function and higher impulsivity (e.g., RVP strength of trace). Note that larger values correspond to worse performance for all but one measure, only within the measure “RVP sensitivity to target” smaller values reflect worse performance. Data were normalised to mean zero and a standard deviation of one.

In a first step, a robust outlier detection was applied on the pre-test data to identify subjects that showed atypical behavioural performance in six parameters of attentional and executive functions. The outlier detection algorithm identified 12 of the population of 35 subjects as outliers with atypical values. The position of these outliers within the multivariate configuration is illustrated in Figure 2A. The outlier detection algorithm performs a robust PCA to identify atypical positions within the multivariate configuration. Figure 2A depicts the position of each subject within the first three PCA components that explain 97 per cent of the variance. The loadings of each behavioural parameter on each PCA component (i.e., the variable weights or eigenvectors) are illustrated in Figures 2D–F. Whereas the first component was mainly a linear combination of measures of executive function with high weights on “IED total errors” and “IED extradimensional set shift errors,” the second component reflected a mix of attentional and executive processing with high weights on “RVP response time latency” and “IED intra-dimensional set shift errors.” The third component showed a strong negative loading on the variable “RVP strength of trace” which assesses the individual tendency to show a high number of false alarms (Frey and Colliver, 1973). Note that this variable correlated with self-reported measures of impulsivity as obtained with the BIS 15 (*r* = −0.50, *p* = 0.002, permutation test; Spinella, 2007; Meule et al., 2011). Subjects with self-reported high impulsivity responded less conservative and showed more responses following low perceptual evidence. To sum up, the subgroup with atypical behavioural performance tend to show extreme PCA scores in the direction of higher cognitive costs for set-shifting, slow response times in a sustained visual attention task and higher impulsivity.

To test the robustness and consistency of the outlier classification, the classification was iteratively repeated with a leave-out-one-sample loop. Almost all subjects were consistently classified as outliers or non-outliers in every classification step. Three non-outliers were classified as outliers in 3 per cent of the repetitions, the remaining subjects were classified as outliers or non-outliers with a consistency of 100 per cent. Only one non-outlier showed an inconsistent classification (see Figures 2A–C; green triangle) and was classified as outlier in only 32 per cent of the repetitions and hence excluded from further analyses leaving 12 outliers and 22 non-outliers.

To show that the applied multivariate outlier detection has higher sensitivity and identified more subjects with extreme performance than a classical univariate approach, an additional analysis was performed in which subjects were classified as outliers if their PCA scores either in the first, second, or third PCA component were located below or above 1.5 standard deviations from the mean. As illustrated in Figure 2G, the multivariate robust outlier detection classified two subjects as outliers who showed no extreme positions in individual univariate distributions of the first, second, or third PCA scores but deviated strongly from the dominant data structure in multivariate space. Figure 2H illustrates that the overall group of outliers in comparison to non-outliers tend to show worse performance in the individual pre-tests. Statistically, outliers and non-outliers significantly differed in four of the six pre-tests, which were IDE extradimensional set shift errors (*p* < 0.001, permutation tests with 5,000 samplings, two-sided), IDE intra-dimensional set shift errors (*p* = 0.03), IED total errors (*p* < 0.001), and RVP response time latency (*p* = 0.02).

In a *post hoc* analysis we tested whether outliers can be segregated from non-outliers by sample characteristics like verbal intelligence (as estimated by the German vocabulary test WST; Schmidt and Metzler, 1992), handedness (Oldfield, 2010), body mass index, age, sex, or working memory (as estimated by the operation span task; Unsworth et al., 2005). Univariate pairwise comparisons did not show any significant difference between outliers and non-outliers. This suggests that the reported cognitive and neural differences in outliers cannot be explained by unspecific sample characteristics unrelated to attentional and executive processing (see Table 1). In an additional analysis we used the five continuous variables of this subset within a multivariate outlier classification (excluding the categorical variable sex). This new analysis identified two outliers from which one outlier overlapped with the original outlier classification; the remaining eleven outliers from the original outlier analysis of attentional or executive functions remained undetected. Hence, the outlier detection based on attentional or executive functions cannot be explained by group differences in age, intelligence, handedness, body mass, or working memory.

**Table 1:** Univariate differences between outliers in verbal intelligence, handedness, body mass index, age, division of sex, and operation span.

	Non-outliers	Outliers	*p*-value
WST intelligence test	32.33 ± 0.49	31.17 ± 0.79	0.22
Edinburgh handedness inventory	86.44 ± 2.75	87.55 ± 3.82	0.81
Body mass index	21.68 ± 0.46	22.6 ± 0.61	0.24
Age in years	23.18 ± 0.59	24 ± 0.78	0.45
Ratio of men	0.41 ± 0.11	0.42 ± 0.15	1.00
Operation span task	58.95 ± 2.33	59.58 ± 2.69	0.88

*Differences in means revealed only non-significant *p*-values. Means ± standard errors of means, *p*-values result from permutation tests*.

### Differences in Behavioural Nicotine Effects Between Non-outliers and Outliers

Following the identification of subjects with extreme behaviour in the attentional and executive pre-tests, we analysed whether outliers and non-outliers show different nicotine effects on attentional distractor costs and executive switch costs (see below for further details). The mean nicotine effect on distractor costs (subtracting distractor costs under nicotine from distractor costs under placebo) significantly differed between non-outliers and outliers (mean nicotine effect on distractor costs for non-outliers: −9 ± 13 ms, mean nicotine effects on distractor costs for outliers: 33 ms ± 13 SEM, *p* = 0.036, two-sample permutation test) showing a significant “distractor costs by drug by outlier group” interaction effect. Further permutation tests revealed a significant mean nicotine effect on distractor costs within the group of outliers (*p* = 0.02), but not within the group of non-outliers (*p* = 0.53).

For switch costs, we found no significant “switch costs by drug by outlier group” interaction effect (mean nicotine effect on switch costs for non-outliers: 13 ms ± 50 SEM, mean nicotine effect on switch costs for outliers: −20 ms ± 65 SEM, *p* = 0.12). Neither the group of non-outliers nor the group of outliers revealed a significant nicotine effect on switch costs (nicotine by switch interaction for non-outliers: *p* = 0.28, outliers: *p* = 0.33). Mean reaction times of each condition can be found in Table 2.

**Table 2:** Mean reaction times in ms and SEMs for outliers and non-outliers as a function of trial type.

	Ongoing trials		Distractor trials		Switch trials	
	Placebo	Nicotine	Placebo	Nicotine	Placebo	Nicotine
Non-outlier	606 ± 18	598 ± 18	768 ± 28	769 ± 32	915 ± 22	894 ± 20
Outlier	665 ± 32	632 ± 26	859 ± 42	792 ± 38	1,050 ± 66	1,038 ± 68

### Differences in Resting-State Brain Network Topology Between Non-outliers and Outliers

**Figure 3:**
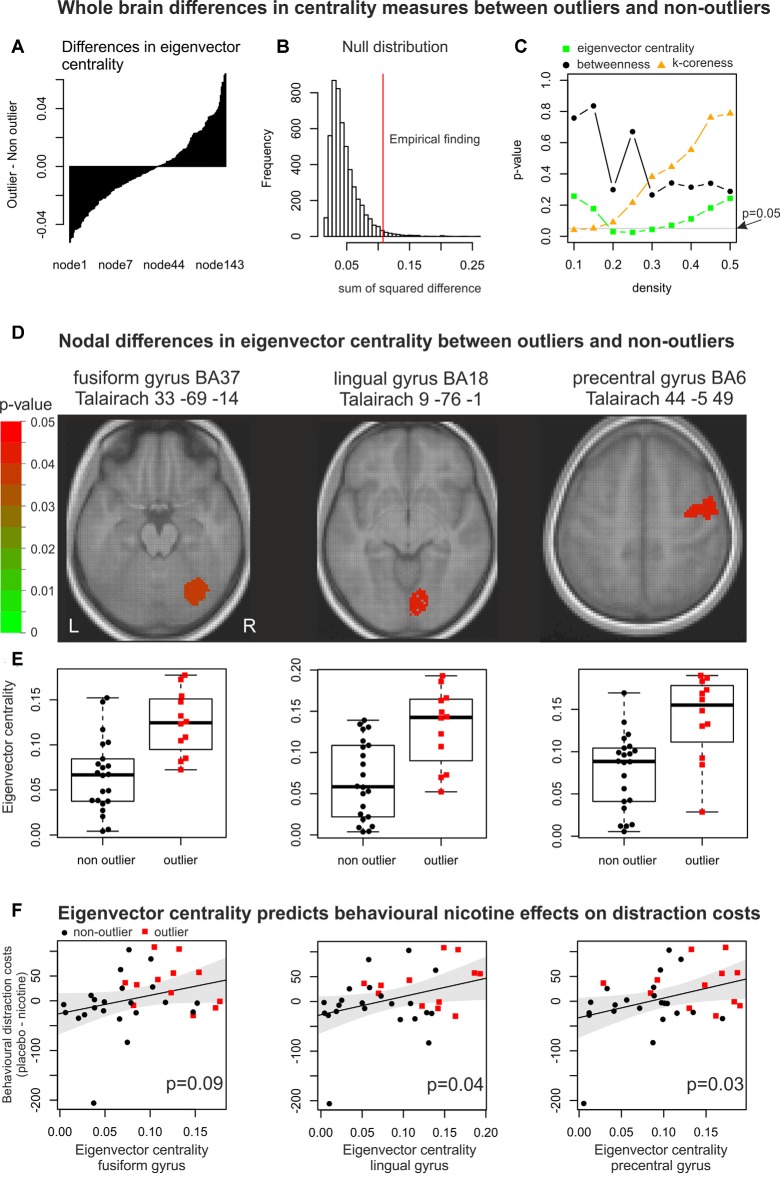
Changes in global and nodal network organisation. Whole-brain differences in centrality between outliers and non-outliers. **(A)** For each node, we computed the mean difference in centrality measures (that is eigenvector centrality, betweenness centrality, and k-coreness) between outliers and non-outliers. The approach is illustrated for the mean differences in eigenvector centrality in brain networks with a network density of 25 per cent, as for this centrality measure and network density the highest significance value (smallest *p*-value) was observed. In a first step, mean differences for each node were sorted from smallest to largest values. This shows that eigenvector centrality increased in some nodes and decreased in others. Thus, for further statistical analyses the sum of squared differences was computed to take into account positive and negative changes. **(B)** In a second step, a randomisation approach in which outliers were randomly shuffled 5,000 times was used to generate a null distribution. The empirical sum of squared differences of eigenvector centrality (indicated by the red vertical line) was unlikely to be found under the assumption that outliers and non-outliers showed no systematic difference. **(C)** The randomisation approach was computed for each centrality measure across network densities. Significant differences were found for eigenvector centrality and k-coreness. Only those network topology measures were analysed node-wise that showed a significant effect on the whole brain level. For these nodal analyses, the network densities with the highest significance values were chosen. Nodal differences in eigenvector centrality between outliers and non-outliers. **(D)** Differences between outliers and non-outliers in eigenvector centrality were found in three brain nodes: the right fusiform gyrus, right lingual gyrus, and right precentral gyrus. For k-coreness no significant nodal differences were observed. **(E)** In these three areas eigenvector centrality was significantly larger for outliers than for non-outliers. Eigenvector centrality predicts behavioural nicotine effects on distractor costs. **(F)** Within two of these three brain regions, the individual values in eigenvector centrality significantly correlated with the behavioural nicotine effects on distractor costs. This points to the fact that larger eigenvector centrality within the right lingual gyrus and right precentral gyrus during resting statepredicted larger behavioural nicotine effects on distractor costs.

The fMRI analysis investigated whether outliers and non-outliers also differed in underlying functional brain network topology gauged by measures of node centrality. Furthermore, we analysed whether these regional differences in network integration predicted the individual behavioural nicotine effects. Since brain networks with the same network density (percentage of connections in comparison towards a fully connected network) were compared, increases in nodal centrality in one brain region might go along with decreases in other regions (see Figure 3A). Therefore, permutation tests were used to test whether the sum of squared nodal differences was significantly larger than expected under the null hypothesis of no difference between outliers and non-outliers (see Figure 3B). The analysis revealed significant differences between outliers and non-outliers for eigenvector centrality, which measures how well a node is connected within the network, at densities of 20, 25, and 30 per cent (see Figure 3C). K-coreness, which is a measure of how efficient a node can spread information within a network, differed between outliers and non-outliers only for networks with 10 per cent network density.

The subsequent nodal analysis was therefore performed for the network density corresponding to the smallest significant *p*-value showing effects on the whole brain level. Hence, nodal effects on eigenvector centrality were analysed with a density of 25 per cent and k-coreness with a density of 10 per cent. Since we did not find significant differences for “betweenness centrality” on a global level (see Figure 3C), we did not investigate it on the nodal level. On nodal level, only eigenvector centrality significantly differed between outliers and non-outliers within the right fusiform gyrus (*p* = 0.037; FDR-corrected), the right lingual gyrus (*p* = 0.045), and the right precentral gyrus (*p* = 0.045; see Figure 3D). Within these brain regions, outliers showed significantly larger eigenvector centrality in comparison to non-outliers (see Figure 3E). K-coreness showed no significant effects on the nodal level. In other words, network integration within visual and premotor brain regions differed between healthy subjects with atypical behavioural and normal performance. Subjects with atypical performance showed an overcompensation in brain network integration.

### Correlation Between Brain Topology and Individual Behavioural Nicotine Effects

In a last crucial step, we asked whether these differences in eigenvector centrality between outliers and non-outliers significantly correlated with the behavioural effects of nicotine on distractor costs (placebo costs minus nicotine costs). The results revealed a significant correlation between distractor costs and eigenvector centrality within the right lingual gyrus (*r* = 0.35, *p* = 0.04, permutation test) and right precentral gyrus (*r* = 0.37, *p* = 0.03) and a trend for a significant effect in the right fusiform gyrus (*r* = 0.30, *p* = 0.09, see Figure 3F).

For each of the brain regions, we performed an additional multiple regression analysis which included the individual maximum framewise displacement, the eigenvector centrality of each subject, and their interaction as independent variables and behavioural effects of nicotine on distractor costs as dependent variable. The results revealed no significant interactions (all *p* > 0.80) which suggest that the association between eigenvector centrality and behavioural nicotine effects is not related to head movements.

## Discussion

The current study aimed to identify subjects that will benefit from a stimulation with the cholinergic agonist nicotine. Our results document that a multivariate outlier detection approach can identify a subgroup of healthy human subjects with atypical patterns of behaviour who differ in resting-state network topology and benefit from nicotine administration. Furthermore, we used complex network analyses to compare different measures of nodal centrality between this subgroup and the remaining population and show that interindividual differences in centrality predict behavioural nicotine effects. Our results provide evidence that the subgroup of outliers shows significantly larger eigenvector centrality within the right fusiform gyrus, the right lingual gyrus, and the right premotor region. Hence, in subjects with atypical performance, these brain regions were more central and more strongly connected. Importantly, larger nodal integration correlated with nicotine-induced improvements in distractibility. In summary, our results suggest that the regional integration of visual and pre-motor areas during rest is a significant predictor of behavioural nicotine effects.

### Robust Outlier Detection in the Multivariate Space

Newhouse et al. (2004) explained the inter-individual variance of nicotine by a one-dimensional inverted U-shape model. Following the Yerkes–Dodson law, cognitive enhancers like nicotine should mainly improve the performance of subjects in a suboptimal performance state with low cholinergic activity (Newhouse et al., 2004). Previous studies that examined baseline dependent effects of pro-cognitive drugs often related cognitive improvements following drug application to *one* pre-existing performance measure in a single cognitive task. For example, univariate baseline-dependent effects of nicotine have been shown for attentional and impulsive behaviour, and executive functions (Thiel et al., 2005; Petrovsky et al., 2012; Potter et al., 2012; Niemegeers et al., 2014; Kasparbauer et al., 2019).

We here provide a new approach to investigate baseline-dependent drug effects. Using multivariate outlier detection with robust statistics we first identified a subgroup of subjects which showed atypical attentional and executive performance in multivariate space and subsequently investigated whether these subjects differed in brain network topology and the behavioural effects of nicotine on attention and executive function. Possibly due to their reduced ability to focus attention and lapses of attention under conditions of high task demands, the most extreme outliers tended to be values in the direction of higher impulsivity and slower response time latencies in a sustained visual attention task (Keilp et al., 2005; Tamm et al., 2012). We demonstrate that the multivariate approach identified subjects as atypical performers that would not have been identified if each variable had been considered independently. Hence, multi-variate approaches show higher sensitivity to identify subgroups of subjects which may not necessarily be characterised by an extreme value in the univariate distributions.

From these atypical subjects, we selected those subjects whose latent scores within the multivariate PCA space pointed towards worse performance or higher impulsivity on at least one latent factor. Future studies with larger samples might use more detailed searchlights to divide this multivariate space into smaller subspaces to even further improve the prediction of individual drug responses. A different approach would be to avoid the dichotomisation of distance scores that each subject shows towards the multivariate centre. Without grouping subjects into outliers and non-outliers, future studies might directly investigate the correlations between distance scores, behavioural nicotine effects, and brain network topology.

The current analysis revealed a relatively large subgroup of outliers. Previous results documented that outlier detection algorithms differ in their hit and false-positive rates to detect outliers and that multivariate robust techniques outperform univariate approaches (Templ et al., 2019). However, further research might provide additional evidence which algorithms are most suitable for small sample sizes (Jones, 2019).

Despite the moderate sample size, we think that the results are valid since our analyses show that the identified subcohort of extreme subjects differs neurobiologically. The subcohort, which was identified solely on behavioural data, showed significant differences in resting-state brain network topology. Furthermore, our results show that these brain regions, which show different network topology, predict the behavioural effects of nicotine if subjects are exposed to the drug in a following pharmacological intervention study.

### The Resting-State Topology Predicts Behavioural Nicotine Effects

The outlier detection approach enabled us to identify those subjects with atypical patterns of performance whose latent scores pointed towards worse attentional or executive performance or higher impulsivity on at least one latent factor. Our analysis revealed that this subgroup is characterised by differences in the integration of the right visual association cortex and pre-motor areas during rest. Increased eigenvector centrality was significantly associated with atypical patterns of attentional, executive, and impulsive behaviour and predicted behavioural nicotine effects within the following experimental sessions. A prior pharmacological fMRI study documented that patterns of local brain activity within the left posterior cingulate cortex, the right superior parietal cortex, the right dorsal medial prefrontal cortex, and the left ventral medial prefrontal cortex significantly predicted behavioural nicotine effects in visual-spatial attention (Giessing et al., 2007). Within one of our previous studies, we also documented that baseline levels of global network topology correlated with the behavioural effects of nicotine: smokers with less integrated brain networks showed the greatest improvements in performance after nicotine replacement (Giessing et al., 2013). Here, we extended this prior work and demonstrated that extreme patterns of cognitive functioning in non-smokers are related to differences in brain network topology which, in turn, predict individual behavioural effects of a later nicotine administration. Possibly, a maladaptation of excessive local brain integration in visual association and pre-motor areas can be reduced by nicotine administration.

In a coordinate-based meta-analysis, Sutherland et al. (2015) reported an impact of nicotine on BOLD activity in the premotor region and lingual gyrus across a wide variety of task-based nicotine neuroimaging studies. However, experimental evidence on whether nicotine is able to decrease eigenvector centrality in visual and motor areas is currently lacking. Two prior studies investigated the effects of an acute administration of nicotine on resting-state brain network topology but focussed on measures of brain network efficiency rather than centrality measures (Wylie et al., 2012; Giessing et al., 2013). Contrary to our explanation, both studies suggested that nicotine increases local and global brain network integration. In addition, Giessing et al. (2013) showed that for smokers attentional improvements following nicotine correlated with drug-induced increases in brain network integration within the right motor cortex. However, there is also supporting evidence that nicotine decreases the connectivity within brain regions involved in motor processing. A pharmacological resting-state electroencephalography (EEG) study provides evidence that, in non-smokers, nicotine decreased connectivity between precentral gyrus, parietal regions, and occipital regions in the upper alpha frequency range (Ranzi et al., 2016).

### Clinical Implications

There is currently a debate whether clinically relevant deviations in behaviour reflect the endpoints of continuous differences rather than distinct categorical conditions (Marcus and Barry, 2011; Stoyanov et al., 2019). If patients with ADHD reflect an extreme of a behavioural spectrum, healthy outliers in attention, executive functions or impulsivity, as identified in this study, might reflect the intermediate position on a dimension between healthy subjects and clinical disorders like ADHD. Recent brain imaging studies emphasised the importance of a network perspective to understand the underlying mechanisms of ADHD (Castellanos and Proal, 2012). Several studies reported, depending on the investigated brain network, increased and decreased functional connectivity in ADHD patients. For example, Tomasi and Volkow (2012) found increased short-range connectivity, but reduced long-range connectivity in ADHD subjects. These findings fit with the results of Lin et al. (2013) showing a reduction of overall network integration in ADHD patients. Many studies in children and adults with ADHD, however, documented significant increases in the connectivity of functional brain networks in comparison to healthy controls (McCarthy et al., 2013; Barber et al., 2015; Sidlauskaite et al., 2016). Cocchi et al. (2012) showed increased connectivity within the right precentral gyrus in a group of non-clinical, drug-naive high-functioning young men and women with ADHD. Qian et al. (2019) documented an increase in connectivity and loss of between-network functional segregation in children with ADHD within primary visual brain regions. In short, consistent with our results, there is previous evidence that patients with ADHD show increased local brain connectivity within visual and pre-motor brain regions supporting a possible maladaptation within these brain regions.

Our findings provide additional evidence that a nicotine-based treatment has the potential to improve attentional performance in a subgroup of predisposed subjects that show atypical patterns of low attentional or executive behaviour and differences in local integration of functional brain network topology. This finding may also be of relevance for other disorders such as mild cognitive impairment where a pilot trial on long term nicotine treatment resulted in improved attention, memory and psychomotor speed (Newhouse et al., 2012) or the treatment of cognitive dysfunction in patients with schizophrenia (Tregellas and Wylie, 2019).

## Conclusion

The current study used six parameters of two behavioural tests to assess individual baseline differences in attention, executive functions, and impulsivity. However, the applied approach is also suitable to incorporate larger data sets with various behavioural tests and questionnaires to characterise the phenotypes of individual subjects. Thus, the presented analyses might encourage future studies with larger sample sizes to replicate our results and to explore the entire multivariate range of individual performance patterns to identify subgroups of healthy subjects or patients with different drug responses and different underlying brain connectivity.

## Data Availability Statement

The datasets generated for this study are available on request to the corresponding author.

## Ethics Statement

The studies involving human participants were reviewed and approved by Ethics Committee of the German Psychological Association (DGPs). The patients/participants provided their written informed consent to participate in this study.

## Author Contributions

CT and SA designed the study and implemented the data acquisition. CG conceptualised and performed the data analysis and took the lead in writing the manuscript with contributions from CT and SA.

## Conflict of Interest

The authors declare that the research was conducted in the absence of any commercial or financial relationships that could be construed as a potential conflict of interest.

## Footnotes

^1^www.cantab.com

^2^https://www.r-project.org/

^3^http://www.mni.mcgill.ca/

^4^https://github.com/asoroosh/DVARS
